# Nutritional Status and Sarcopenia in Nursing Home Residents: A Cross-Sectional Study

**DOI:** 10.3390/ijerph192417013

**Published:** 2022-12-18

**Authors:** Nan Hua, Yifan Zhang, Xiangmin Tan, Li Liu, Yihan Mo, Xuemei Yao, Xiuhua Wang, James Wiley, Xiaoqing Wang

**Affiliations:** 1 Xiangya School of Nursing, Central South University, Changsha 410013, China; 2 School of Life Sciences, Central South University, Changsha 410013, China; 3 Cicely Saunders Institute of Palliative Care, Policy & Rehabilitation, King’s College London, London SE5 9PJ, UK; 4 Tangdu Hospital, Air Force Military Medical University, Xi’an 710032, China; 5 School of Nursing, University of California, San Francisco, CA 94118, USA; 6 Department of Geriatrics, The Second Xiangya Hospital, Central South University, 139 Renmin Middle Road Furong District, Changsha 421142, China

**Keywords:** older adults, nutrition, sarcopenia, nursing homes, risk factors

## Abstract

Objective: This study aimed to assess the nutritional status and sarcopenia in older people living in nursing homes. Methods: This cross-sectional study enrolled 386 older adults in nursing homes in Hunan Province, China. Assessments included the Mini Nutritional Assessment Short Form for nutrition risk, Dietary Diversity Score for dietary diversity and Mini Mental State Examination for cognitive status. Sociodemographic (e.g., age, sex and educational level), health-related characteristics (e.g., food intake, self-care status and medication), body composition (e.g., body mass index [BMI], protein, body fat mass [BFM], percent body fat [PBF], skeletal muscle index [SMI] and total body water [TBW]) and anthropometric parameters data (e.g., calf circumference [CC], upper arm circumference [UAC], handgrip and gait speed) were also collected. Malnutrition and their associated risk were analyzed by multivariable Poisson regression analysis. Results: In total, 32.4% of participants (*n* = 125) were at risk of malnutrition and 49.7% (*n* = 192) suffered from sarcopenia. Nutritional status was positively associated with age (risk ratio [RR] = 1.03), sarcopenia (RR = 1.88), tooth loss affecting food intake (RR = 1.45), low self-care status (RR = 1.82) and moderate/inadequate dietary diversity (RR = 2.04) and negatively associated with one child (RR = 0.27), BMI (RR = 0.82), protein (RR = 0.76), BFM (RR = 0.91), PBF (RR = 0.94), SMI (RR = 0.65), TBW (RR = 0.94), CC (RR = 0.89) and UAC (RR = 0.86). Conclusions: Age, number of children, sarcopenia, food intake, self-care status, dietary diversity and body composition were associated with malnutrition among nursing home residents. For vulnerable groups, researchers should focus on raising the body composition indicators, such as BMI, protein, BFM, SMI and TBW and measuring CC and UAC for initial screening.

## 1. Introduction

Aging has become a global social issue and an inevitable trend in modern society. According to the World Health Organization prognosis [1], the population aged 60 and over is projected to account for up to 22% of the entire population in 2050. China, as the country with the largest older population in the world, is expected to have an older population of over 400 million in 2050, the population aging coefficient may exceed 30% and the older people dependency ratio continuously increases [2].

Older people are more likely to consume insufficient amounts of food and dietary nutrients because of their reduced basal metabolic rates [3], chewing and swallowing impairment [4], swallowing [5], digestion and absorption capacities [6] and lower levels of physical activity [7]. Malnutrition in older adults can lead to several adverse clinical outcomes, such as increased disability, morbidities, mortality rate and deteriorated quality of life [8,9,10,11]. Malnutrition or the presence of nutritional risks can also increase the incidence of falls, osteoporotic fractures and hospital-acquired infections [12,13]. Diet and nutrition have become major drivers of global chronic disease morbidity [11]. The overall cost of treating diseases caused by malnutrition in 2012 was 84.14 billion yuan [14]. Malnutrition also resulted in an increase of 32% in annual per capita hospitalizations and a 31% increase in annual per capita hospitalization costs among older people in China in 2015 [15], thus increasing the treatment costs by 45.84 billion yuan and causing a significant financial strain on families and society [16,17]. Sarcopenia is a geriatric syndrome, characterized by age-related muscle loss, poor muscle strength and/or decreased physical performance [18]. The number of people suffering from sarcopenia is expected to reach 1.2 billion by 2025 and more than double by 2050 [19]. Sarcopenia is related with negative health outcomes such as an increased risk of falls, frailty, hospitalization, morbidity and death [20].

In China, more than two million senior citizens choose to live out their senior years in nursing homes because they are neglected or suffer from severe chronic illnesses that require specialized medical care [21]. In comparison with other older adults who can take care of themselves in the community, nursing home patients may be in a more nutritionally vulnerable condition. In recent years, the nutritional status of older people in nursing homes has received worldwide attention. The results of multicenter prospective studies showed that the rates of malnutrition among older adults in hospitals, communities and nursing homes were 38.7%, 5.8% and 17.5%, respectively [22,23]. Considering the different participant selections (e.g., whether to include people with severe cognitive impairment) and screening or evaluation methods (e.g., questionnaire assessments, anthropometric measurements and body composition analysis), the prevalence of malnutrition among older adults in nursing homes varies and ranges from 8.2% to 30% [9,24,25]. Besides, declining health status in older adults is associated with changes in body composition [26] and muscle mass loss is regarded as an early identifier of adverse changes in body composition [27]. However, the association between nutritional status and body composition of nursing home residents in Hunan Province remains unknown. Understanding the nutritional status of nursing home residents and associated factors can help in the development of preventative healthcare strategies and improve dietary health management in nursing homes. Few investigations have focused on nutritional status and characteristics related to different health-related performance, body composition, sarcopenia and dietary diversity, despite the growing number of older individuals at risk of malnutrition among nursing home residents. The relationship between nutrition risk and health-related performance and body composition needs to be determined for the development of effective interventions.

Therefore, this study aims to assess the nutritional status and sarcopenia in nursing home residents and to explore its associated factors, in order to provide a basis for future research on nutritional interventions for older adults in nursing homes and thus promote healthy aging.

## 2. Methods and Materials

### 2.1. Study Design and Participants

This cross-sectional study was conducted from June to November 2021 and the studied group consisted of 386 older adults from 17 nursing homes in Hunan Province, China. According to the 17.5% prevalence of malnutrition in Chinese nursing home residents [23], the minimum sample size required for this study was 226, at 5% significance, 80% power and 20% dropout rate, as assessed by University Dusseldorf G*Power 3.1 software. We divided Hunan province into five parts (east, west, south, north and central) according to geographic orientation, then randomly selected nursing homes from each of the five regions and recruited older people who met the criteria of the study. The inclusion criteria were as follows: (1) age ≥60 years; (2) residency in a nursing home for at least 2 months; (3) conscious and ability to understand and cooperate in completing questionnaires and various tests; and (4) signed an informed consent form (signed by participants or their legally authorized proxies). The exclusion criteria were as follows: (1) with implanted pacemakers; (2) with organ failure; and (3) with prostheses.

### 2.2. Data Collection

Face-to-face investigation, body composition and physical performance assessments were conducted to participants by trained researchers. The questionnaire used included effective tools for evaluation of malnutritional risk, (Mini Nutritional Assessment Short Form [MNA-SF]), dietary diversity, (Dietary Diversity Score [DDS]) and cognitive status (Mini Mental State Examination [MMSE]). Sociodemographic information was obtained from participants and other information including self-care status, use of medication, food intake and comorbidities were obtained from medical files and staff.

### 2.3. Assessments

#### 2.3.1. Risk of Malnutrition

MNA-SF questionnaire was used to assess nutritional status [28], according to recommendation by the Chinese Medical Association Geriatric Nutrition Support Group for older patients [29]. It has been widely used in practice to screen the risk of malnutrition [30]. It includes the body mass index (BMI), weight loss, recent stress or illness, mobility, cognitive function and dietary status. The Cronbach’s alpha coefficient was 0.711 [31] and nutritional status was defined as normal nutritional status (score 11–14), at risk of malnutrition (score 8–10) and malnourished (score < 8), based on the suggested cutoff points for greater specificity [30].

#### 2.3.2. Anthropometric Characteristics and Body Composition

All anthropometric procedures were performed by qualified specialists and standardized criteria were used to ensure reliability. BMI was determined by dividing the weight (kg) by the square of the height (m). Height was measured using a smart instrument fixed to the wall (Yolanda CH10A; Shenzhen, China). Weight was measured using a calibrated scale (MI ZC01HM; Hefei, China). Calf circumference (CC) and upper arm circumference (UAC) were measured using a millimeter-rated non-elastic tape measure without compressing the subcutaneous tissues. The bioelectrical impedance analysis (BIA) device (Inbody S10; Biospace, Seoul, Korea) was used to measure body composition including protein, body fat mass (BFM), percent body fat (PBF), minerals, skeletal muscle index (SMI) and total body water (TBW).

#### 2.3.3. Muscle Mass

The muscle mass was accessed via BIA. Participants were required to fast for 2 h before the measurement to ensure an empty urinary bladder and lack of residual stool during the measurement. Muscle mass was expressed as SMI, with SMI < 7.0 kg/m^2^ in male and <5.7 kg/m^2^ in female indicating low muscle mass [32].

#### 2.3.4. Muscle Strength

The muscle strength was evaluated using a digital hand dynamometer (EH101; Guangzhou, China). The higher value was reported while testing the dominant hand’s grip strength two times. Male with a grip strength lower than 28 kg and female with a grip strength lower than 18 kg indicated low muscle strength [32].

#### 2.3.5. Physical Performance

A 6-m gait speed test was used to assess physical performance [32]. A stopwatch was used to monitor the time taken by the subjects while they walked 6 m at their normal pace. A cut-off point of <1.0 m/s indicated low physical performance [32].

#### 2.3.6. Sarcopenia

Sarcopenia was diagnosed according to the consensus of the Asian Working Group for sarcopenia [32]; low muscle mass, low muscle strength with or without low physical performance were identified as sarcopenia.

#### 2.3.7. Dietary Diversity

The dietary diversity was evaluated via DDS, which keeps track of nine food groups including cereals, vegetables, fruits, legumes, eggs, poultry, fish and shrimp, dairy products, fats and oils. It assigns a score based on the types of foods consumed throughout the week, in which one point was allocated to each food item consumed and a maximum of nine points can be given. This approach is a reliable indicator of dietary diversity and nutritional quality [33].

#### 2.3.8. Cognitive Status

MMSE [34] was cross-culturally modified to a Chinese version [35]. It was used to assess the cognitive function of older people and consists of 30 items in five areas: orientation, memory, attention, numeracy and language skills, with a total score between 0 and 30, the higher the score, the better the cognitive function. The MMSE score ≤ 24 indicates impaired cognitive function and Cronbach’s alpha coefficient was 0.91 [35].

### 2.4. Ethics Statement

Ethical approval for this study was obtained from the Central South University Ethics Commission (E2021132). The study commenced after the facility managers and older people have been informed in detail of the study’s purpose, content and process and after they signed the informed consent form (signed by participants or their legally authorized proxies).

### 2.5. Statistical Analyses

According to the data type and parametric test assumptions, the findings were expressed as the median (quartile 1) and number-percentage. Kolmogorov-Smirnov test was used to check the normal distribution of continuous variables, while the χ^2^ test was employed to determine differences between categorical variables. Mann-Whitney U test was used in the absence of parametric test hypothesis for quantitative variables. Statistical significance was considered at *p* < 0.05 for all tests. The risk ratios (RRs) and 95% confidence intervals (95% CIs) were used to assess the factors that contribute to nutrition risk. SPSS Statistics (IBM 26.0) software was used to analyze the collected data.

## 3. Results

### 3.1. Characteristics of Participants

The sociodemographic characteristics, health-related and anthropometric characteristics are described in Table 1. A total of 386 older adults were included, with a sex ratio M/F of 0.76. The mean age was 80.55 ± 8.96, which was lower for males than in females (79.43 ± 0.77 vs. 80.97 ± 0.58 years; *p* < 0.001). Of the total sample, nearly half of participants are aged between 80 and 89 and only received primary level education. More than half of 195 of older adults were in divorced or widowed. Most of the participants (79.7%) had resided in the nursing home for less than three years. Although 71.5% of participants had two or more children, only 31.9% had their children or grandchildren cover the costs of their pensions and the majority (53.9%) of older people have their pensions provided by themselves or spouses. Nearly half of the participants have lost teeth that affecting food intake and 27.2% are unable to provide care for themselves completely. Almost three-quarters (72%) of older people are receiving medication. In total, 90.4% of older persons have adequate dietary diversity, in which 45% of them have a cognitive impairment and one in 10 participants (11.7%) have three or more comorbidities (e.g., cardiovascular disease, respiratory disease, endocrine disease and other chronic diseases such as osteoporosis and arthritis). No significant variations were observed in the frequency of older adults with normal muscle mass and frequency with reduced muscle mass. Besides, only about 10% of participants had normal muscle strength and gait speed. All anthropometric measurements were lower in the malnutrition risk group compared with the normal nutrition group (*p* < 0.001).

### 3.2. Nutritional Status and Sarcopenia

The proportion at risk of malnutrition or malnourished was 32.4%. Among those with moderate or insufficient dietary diversity, 64.9% were at risk of malnutrition or malnourished. In total, 75 (39.9%) people experience tooth loss that affects intake and 53 (50.5%) people cannot fully provide care for themselves and are at risk of malnutrition or malnourished. The results indicate that high BMI is advantageous for nutritional status. The prevalence of sarcopenia was 49.7%. Moreover, higher body composition markers, such as protein, BFM, PBF, minerals, SMI and TBW, are associated with lower nutritional risk. Nutritional status had no significant association with medication, comorbidities and cognitive status.

### 3.3. Factors Associated with Nutritional Risk

Poisson regression with robust variance was performed to identify possible nutritional risk factors and the results are shown in Table 2. For the robust Poisson regression model, after adjusting all potential confounding factors, the nutritional risk was positively associated with age, sarcopenia, tooth loss affecting food intake, low self-care status and moderate/inadequate dietary diversity and negatively associated with one child, BMI, protein, BFM, PBF, SMI, TBW, CC and UAC.

## 4. Discussion

Older adults become more prone to malnutrition as they age, leading to immunity and bacterial resistance, increased susceptibility to illness, premature frailty and aging and deteriorated quality of life [10,36]. Older adults with a “fair” self-rated nutritional status had a 46% higher mortality rate than those with “at least good” [37]. The nutritional condition needs to be determined and the associated risk factors that contribute to the malnutrition of nursing home residents need to be identified to prevent chronic diseases, promote health and raise the quality of life.

Malnutrition is a common health predicament among older adults. Based on MNA-SF results, the proportion of older people in nursing homes at risk of malnutrition was 32.4%. The nutritional status of nursing home residents in different regions of China was in the range of 33.9–52.4% in terms of prevalence of malnutrition [15,38]. A multicenter prospective study conducted by the International MNA Assessment Organization showed that the prevalence of malnutrition in nursing homes was 38.7% [22]. Therefore, malnutrition among older people is a global problem, which is related to the level of economic development, aging degree and residency. The percentage in our study is considerably lower than that found in the above-mentioned study. Firstly, this finding can be attributed to the development of the economic level, in which nursing homes pay great attention to nutrition, with a variety of food that is easy to be digested and meet the dietary recommendations, thus improving the nutritional status. Secondly, considering the protection of vulnerable groups by nursing home staff, such as those suffering from serious diseases, psychological disorders, poor family support and low cooperation, relevant data cannot be accessed, although these people are likely to be nutritionally substandard.

In multinomial analysis, age is an independent risk factor for nutritional risk. The progressive decline of bodily condition caused by aging, loss of teeth and the decrease in the number of taste buds affect food selection and intake [39,40]. However, in the Chinese context, older people are more frugal in their diet and pay less attention to nutrition. As a result of advancing infirmity, people that do not receive pension have no reliable source of income other than government subsidies or financial support from their children, thus limiting their ability to access adequate nutrition and complicates composition of a balanced diet. More importantly, aging increases the susceptibility of older people to diseases that deplete the body’s nutritional reserves [41].

In terms of sociological factors, the ratio of nutritional risk was 35.2% in the female population. No association was found between nutritional risk and gender, marital status and educational level. However, a recent study of institutionalized older people revealed that female residents had lower energy and nutrient intakes and were more likely to be malnourished than male [42]. No statistically significant association was observed between gender and nutritional risk in the results of the univariate analysis, although women showed a greater incidence of nutritional risk as in other studies. Highly educated older persons are more conscientious about health and are more capable of acquiring nutritional knowledge and taking an active role in developing healthy eating habits and nutritional behaviors [43,44]. Another study has shown that marital status is negatively associated with the nutritional status of older adults, in which those in the widowed or divorced group have lower nutritional status than those in the spoused group [45]. In this study, the education level of nursing home residents did not reveal a significant trend in terms of nutritional risk and residents who were married had a lower nutritional risk (24.8%) than single, divorced, or widowed participants.

Family involvement and contact are the primary forms of interpersonal interaction for older adults. However, those in nursing homes have fewer opportunities to acquire social support, because they have no relatives or children. Social support can influence older adults’ self-efficacy and actions that promote health in a beneficial way [46]. According to the present study, older people without children are likely to experience malnutrition because of the lack of social support. The residence times and source of pension are unique demographic characteristics of nursing homes and do not affect the risk of malnutrition, consistent with another study [47].

The number of comorbidities had no effect on the nutritional status. This finding was obtained because some older adults have only one comorbidity, but this disease is more serious and remarkably affects appetite and gastrointestinal function. This condition restricts the intake of certain diets during the treatment process, leading to malnutrition caused by insufficient nutrient intake. Second, some older adults have multiple chronic diseases but their conditions are better controlled and their nutritional status may be the same as that of normal non-diseased older people. Further study will be needed to confirm this. In conclusion, the relationship between nutritional risk and the number of comorbidities was not significant without analysis of confounding factors. In addition, older adults who take multiple oral medications are likely to experience malnutrition caused by the side effects [48]. Older adults with chronic conditions experience prolonged pain and bear the cost of expensive tests, treatments and medications, which can lead to negative emotion and decreased appetite and multiple medications can directly or indirectly affect nutrient intake, absorption and metabolism, resulting in the development of malnutrition. However, similar to the findings of Smoline et al. [49], we found no association between medication use and risk of malnutrition, which may be related to the small proportion of multiple medication use in this sample.

The results suggest that one of the key determinants of nutritional status is the effect of tooth loss on diet. Surprisingly, almost as many people had eating problems caused by tooth loss as did not, probably because the nursing home provided a similar amount of nutritional supply with the help of denture wear and assisted feeding. We found that, in the group with tooth loss affecting intake, the nutritional status was significantly affected (1.80 times higher risk of malnutrition compared to older people who eat normally, potential confounding factors were controlled). Dietary diversity is an important indicator for assessing the nutritional quality of a diet [50] and DDS is a commonly used quantitative tool to assess the dietary diversity and the nutritional quality of a diet. A rich and varied diet is an independent protective factor for nutritional status and the majority of older people in this sample had an adequate variety of diets because of the improved living standards.

Muscle mass, muscle strength and physical performance reflect the functional state of the body’s muscles and whether a person is diagnosed with sarcopenia also relies on these three indicators [32]. Half of participants had low muscle mass and a higher percentage corresponded with low muscle strength and low gait speed (88.9% and 87.8%, respectively). Muscle strength, as measured by handgrip strength, is a marker of nutritional status. Surprisingly, no association was observed between low muscle strength and nutritional risk, which is consistent with previous reports [51]. The grip strength meter itself may be subject to measurement and display errors, as it is specified that a force of 1.4 kg to 1.8 kg is required to produce accurate readings [52], even though the grip strength meter is considered to be the best tool for measuring muscle strength. Additionally, older people with low muscle mass are more likely to experience malnutrition. Since older adults who experience malnutrition typically lose weight and skeletal muscle, this results in severely decreased muscle strength and function [3]. Sarcopenia makes frail older adults worse off and further aggravates physical health problems. Therefore, nursing home residents who are malnourished or even at risk of malnutrition need to be alerted about the possibility of sarcopenia, with timely nutritional therapy is needed to reduce nutritional risk and thus improve the clinical outcome of patients. Consistent with previous studies, BMI, protein, BFM, PBF, minerals, SMI, TBW, CC and UAC are all valid predictors of malnutrition and these easily accessible data provide caregivers with additional options for measuring the nutritional status or assessing nutrition risk.

Considering the growing pace of aging and the susceptibility of nursing home residents, nearly one-third of older persons are in danger of malnutrition, making nutrition a severe public health concern.

## 5. Strengths and Limitations

The strength of this study is that it explores the health status of older adults in nursing facilities at different levels of health, considering the effect of body composition and muscle health on nutrition. Our research employed a widely-used scientific tool for assessing nutritional status (MNA-SF). The limitation is that some factors that influence nutritional status were not measured in our retrospective cross-sectional study and should be included in subsequent work. In the absence of controlled experiments or longitudinal data, we can only report correlations and only speculate about causality. More follow-up research is needed in the future with an extended observation period, more complete measurement of risk factors for malnutrition and a larger more diverse sample.

## 6. Conclusions

The findings suggest that malnutrition is common among nursing home residents. Room style, muscle mass, self-care status, dietary diversity, diet and protein were identified as relevant risk factors influencing the nutritional risk of nursing home residents. Screening facilitates the early identification of nutritional risk sensitive groups and particular attention should be paid to body composition indicators such as BMI, protein, BFM, SMI and TBW of nursing home residents. Simple CC and UAC measurements also need to be carried out. Future interventions should focus on manageable and easily improved aspects to prevent or delay the onset of malnutrition as much as possible.

## Figures and Tables

**Table 1:** Participants’ characteristics by nutritional status.

Sociodemographic Characteristics	Total, *n* (%)	Nutrition Risk Status		*p* Value
		Normal Nutritional Status, *n* (%)	At risk of Malnutrition or Malnourished, *n* (%)	
All participants	386	261 (67.6)	125 (32.4)	
Sex				ns ^a^
Male	167 (43.3)	119 (45.6)	48 (38.4)	
Female	219 (56.7)	142 (54.4)	77 (61.6)	
Age				0.043 ^a^
60–69	55 (14.2)	43 (16.5)	12 (9.6)	
70–79	94 (24.4)	66 (25.3)	28 (22.4)	
80–89	181 (46.9)	122 (46.7)	59 (47.2)	
≥90	56 (14.5)	30 (11.5)	26 (20.8)	
Educational level				ns ^a^
Primary	182 (47.2)	114 (43.7)	68 (54.4)	
Secondary	163 (42.2)	119 (45.6)	44 (35.2)	
Tertiary	41 (10.6)	28 (10.7)	13 (10.4)	
Marital status				ns ^a^
Single	32 (8.3)	21 (8.0)	11 (8.8)	
Married	101 (26.2)	76 (29.1)	25 (20.0)	
Divorced/Widowed	253 (65.5)	164 (62.8)	89 (71.2)	
Time living in the nursing home (year)				ns ^a^
<1	143 (37.0)	97 (37.2)	46 (36.8)	
1–3	165 (42.7)	104 (39.8)	61 (48.8)	
4–6	25 (6.5)	20 (7.7)	5 (4.0)	
>6	53 (13.7)	40 (15.3)	13 (10.4)	
Number of children				0.008 ^a^
None	55 (14.2)	31 (11.9)	24 (19.2)	
1	55 (14.2)	46 (17.6)	9 (7.2)	
≥2	276 (71.5)	184 (70.5)	92 (73.6)	
Sources of pensions				ns ^a^
Self/Spouse	208 (53.9)	147 (56.3)	61 (48.8)	
Children/Grandchildren	123 (31.9)	81 (31.0)	42 (33.6)	
Government/Collective	55 (14.2)	33 (12.6)	22 (17.6)	
Living Arrangements				ns ^a^
Single room	186 (48.2)	123 (47.1)	63 (50.4)	
Multi-bed room	200 (43.3)	138 (52.9)	62 (49.6)	
Health-related and anthropometric characteristics	*n* (%)	*n* (%)	*n* (%)	*p* value
Tooth loss affects food intake				0.002 ^a^
Yes	188 (48.7)	113 (43.3)	75 (60.0)	
No	198 (51.3)	148 (56.7)	50 (40.0)	
Self-care status				<0.001 ^a^
Completely self-care	281 (72.8)	209 (80.1)	72 (57.6)	
Need help	105 (27.2)	52 (19.9)	53 (42.4)	
Medication				ns ^a^
Yes	278 (72.0)	190 (72.8)	88 (70.4)	
No	108 (28.0)	71 (27.2)	37 (29.6)	
Number of Comorbidities				ns ^a^
<3	341 (88.3)	231 (88.5)	110 (88.0)	
≥3	45 (11.7)	30 (11.5)	15 (12.0)	
Cognitive status				ns ^a^
Normal	211 (54.7)	151 (57.9)	60 (48.0)	
Abnormal < 24	175 (45.3)	110 (42.1)	65 (52.0)	
Dietary diversity				<0.001 ^a^
Adequate	349 (90.4)	248 (95.0)	101 (80.8)	
Moderate/insufficient	37 (9.6)	13 (5.0)	24 (19.2)	
Sarcopenia				<0.001 ^a^
Yes	192 (49.7)	153 (58.6)	41 (32.8)	
No	194 (50.3)	108 (41.4)	84 (67.2)	
Muscle mass				<0.001 ^a^
Healthy muscle mass	191 (49.5)	150 (57.5)	41 (32.8)	
Low muscle mass	195 (50.5)	111 (42.5)	84 (67.2)	
Muscle strength				ns ^a^
Healthy muscle strength	43 (11.1)	34 (13.0)	9 (7.2)	
Low muscle strength	343 (88.9)	227 (87.0)	116 (92.8)	
Physical performance				0.006 ^a^
Healthy gait speed	47 (12.2)	40 (15.3)	7 (5.6)	
Low gait speed	339 (87.8)	221 (84.7)	118 (94.4)	
	Median (IQR)	Median (IQR)	Median (IQR)	*p* value
BMI (kg/m^2^)	23.57 (3.9)	24.10 (3.30)	21.30 (4.40)	<0.001 ^b^
Protein (kg)	7.20 (1.49)	7.30 (1.80)	6.40 (1.70)	<0.001 ^b^
BFM (kg)	20.33 (7.18)	21.60 (7.95)	16.40 (8.05)	<0.001 ^b^
PBF (%)	34.69 (7.50)	35.90 (8.85)	31.90 (10.40)	<0.001 ^b^
Minerals (kg)	2.60 (0.46)	2.63 (0.60)	2.41 (0.53)	<0.001 ^b^
SMI (kg/m^2^)	6.21 (1.06)	6.40 (1.40)	5.60 (1.50)	<0.001 ^b^
TBW (L)	27.30 (7.00)	28.00 (7.00)	24.70 (7.00)	<0.001 ^b^
CC (cm)	33.44 (4.10)	34.00 (3.52)	31.70 (5.07)	<0.001 ^b^
UAC (cm)	26.67 (3.63)	27.25 (3.35)	25.35 (3.43)	<0.001 ^b^

Abbreviations: BMI, Body mass index; BFM, Body fat mass; PBF, Percent body fat; SMI, Skeletal muscle index; TBW, Total body water; CC, calf circumference; UAC, upper arm circumference; IQR, interquartile range. Median (quartile 1) is presented for data not normally distributed and percentages are presented for categorical data. ^a^ χ^2^ test. ^b^ Mann-Whitney U test.

**Table 2:** Factors associated with nutritional risk: univariate and multiple Poisson regression analyses.

Subgroup	Crude RR (95% CI)	*p* Value	Adjusted RR (95% CI)	*p* Value
Age	1.02 (1.01–1.04)	0.010	1.03 (1.01–1.05)	0.008
Number of children				
None				
1	0.38 (0.19–0.73)	0.004	0.27 (0.13–0.55)	<0.001
≥2	0.76 (0.54–1.08)	ns	0.44 (0.25–1.04)	ns
Sarcopenia				
No				
Yes	2.07 (1.51–2.84)	<0.001	1.88 (1.35–2.62)	<0.001
Muscle mass				
Healthy muscle mass				
Low muscle mass	2.01 (1.46–2.75)	<0.001	1.85 (1.33–2.57)	<0.001
Physical performance				
Healthy gait speed				
Low gait speed	2.34 (1.16–4.70)	0.017	1.74 (0.84–3.61)	ns
Tooth loss affecting food intake				
No				
Yes	1.58 (1.17–2.13)	0.003	1.45 (1.06–1.97)	0.018
Self-care status				
Completely self-care				
Need help	1.97 (1.50–2.59)	<0.001	1.82 (1.36–2.44)	<0.001
Dietary diversity				
Adequate				
Moderate/insufficient	2.24 (1.68–2.99)	<0.001	2.04 (1.50–2.78)	<0.001
BMI	0.81 (0.78–0.85)	<0.001	0.82 (0.78–0.86)	<0.001
Protein	0.77 (0.69–0.87)	<0.001	0.76 (0.64–0.90)	0.001
BFM	0.91 (0.89–0.94)	<0.001	0.91 (0.89–0.94)	<0.001
PBF	0.96 (0.94–0.97)	<0.001	0.94 (0.92–0.96)	<0.001
Minerals	0.61 (0.41–0.90)	0.013	0.77 (0.47–1.25)	ns
SMI	0.67 (0.58–0.76)	<0.001	0.65 (0.54–0.77)	<0.001
TBW	0.94 (0.91–0.97)	<0.001	0.94 (0.89–0.98)	0.003
CC	0.88 (0.84–0.92)	<0.001	0.89 (0.85–0.94)	<0.001
UAC	0.85 (0.80–0.89)	<0.001	0.86 (0.82–0.91)	<0.001

Abbreviations: RR, risk ratio; CI, confidence interval; BMI, Body mass index; BFM, Body fat mass; PBF, Percent body fat; SMI, Skeletal muscle index; TBW, Total body water; CC, calf circumference; UAC, upper arm circumference.Robust Poisson model adjusted for sociodemographic factors: age (continuous variable), gender, marital status (×3 categories), education level (×3 categories), living arrangements (×2 categories), number of children (×3 categories) and sources of pensions (×3 categories).

## Data Availability

The data generated during the present study are available from the corresponding author upon reasonable request.
